# Microbial community interactions determine the mineralization of soil organic phosphorus in subtropical forest ecosystems

**DOI:** 10.1128/spectrum.01355-23

**Published:** 2024-02-09

**Authors:** Chang Pan, Chenchen Sun, Xinjing Qu, Wenruinan Yu, Jiahuan Guo, Yuanchun Yu, Xiaogang Li

**Affiliations:** 1 College of Ecology and Environment, Nanjing Forestry University, Nanjing, China; 2 School of Life Sciences, Anqing Normal University, Anqing, China; 3 Co-Innovation Center for Sustainable Forestry in Southern China, Nanjing Forestry University, Nanjing, China; The University of Texas at San Antonio, San Antonio, Texas, USA

**Keywords:** phosphorus stress, organic phosphorus, phosphorus-mineralizing microorganism, Chinese fir, afforestation

## Abstract

In subtropical forest ecosystems with few phosphorus (P) inputs, P availability and forest productivity depend on soil organic P (Po) mineralization. However, the mechanisms by which the microbial community determines the status and fate of soil Po mineralization remain unclear. In the present study, soils were collected from three typical forest types: secondary natural forest (SNF), mixed planting, and monoculture forest of Chinese fir. The P fractions, Po-mineralization ability, and microbial community in the soils of different forest types were characterized. In addition, we defined Po-mineralizing taxa with the potential to interact with the soil microbial community to regulate Po mineralization. We found that a higher labile P content persisted in SNF and was positively associated with the Po-mineralization capacity of the soil microbial community. *In vitro* cultures of soil suspensions revealed that soil Po mineralization of three forest types was distinguished by differences in the composition of fungal communities. We further identified broad phylogenetic lineages of Po-mineralizing fungi with a high intensity of positive interactions with the soil microbial community, implying that the facilitation of Po-mineralizing taxa is crucial for soil P availability. Our dilution experiments to weaken microbial interactions revealed that in SNF soil, which had the highest interaction intensity of Po-mineralizing taxa with the community, Po-mineralization capacity was irreversibly lost after dilution, highlighting the importance of microbial diversity protection in forest soils. In summary, this study demonstrates that the interactions of Po-mineralizing microorganisms with the soil microbial community are critical for P availability in subtropical forests.

**IMPORTANCE:**

In subtropical forest ecosystems with few phosphorus inputs, phosphorus availability and forest productivity depend on soil organic phosphorus mineralization. However, the mechanisms by which the microbial community interactions determine the mineralization of soil organic phosphorus remain unclear. In the present study, soils were collected from three typical forest types: secondary natural forest, mixed planting, and monoculture forest of Chinese fir. We found that a higher soil labile phosphorus content was positively associated with the organic phosphorus mineralization capacity of the soil microbial community. Soil organic phosphorus mineralization of three forest types was distinguished by the differences in the composition of fungal communities. The positive interactions between organic phosphorus-mineralizing fungi and the rest of the soil microbial community facilitated organic phosphorus mineralization. This study highlights the importance of microbial diversity protection in forest soils and reveals the microbial mechanism of phosphorus availability maintenance in subtropical forest ecosystems.

## INTRODUCTION

Phosphorus is a limiting nutrient for plant growth globally (1), and this limitation is particularly serious in subtropical regions, where soils are generally strongly weathered (2, 3). In subtropical forests, accumulated organic P (Po) accounts for up to 80% of the total P pool and is the most important potential source of available P after microbial mineralization (4–7). Consequently, elucidating the mechanisms of Po mineralization is fundamental for maintaining the stability and productivity of subtropical forest ecosystems (2, 8–10).

Multiple regulatory factors determine the complex and variable characteristics of the Po pool and mineralization in forest ecosystems. Soil properties such as Fe and Al oxide content influence Po forms by adsorbing Po (11) or soil pH value modifies Po solubility (12, 13). In forest soil Po pools, soil Po accumulation and transformation are largely determined by aboveground vegetation types, litter biomass and quality, and fine root turnover (14–16). However, both aboveground vegetation and soil properties impact members of the soil microbial community, which are associated with soil P availability (17–19). Especially, for soil microbial phosphatases with a contribution of up to 90% of Po mineralization, this critical process controls the dynamics of soil available P (20–22). In subtropical forest ecosystems with lower soil pH, nevertheless, soil Po mineralization is dominated by acid phosphatases (ACPs) (23) encoded by a small number of soil microorganisms, including some saprophytic fungi, ectomycorrhizal fungi, and Proteobacteria (24, 25). However, data on how microorganisms with traits of metabolizing phosphatases regulate soil Po mineralization in forest ecosystems are limited, especially at their community level.

The abundance and diversity of microorganisms associated with soil Po mineralization are generally low (7); consequently, they are susceptible to environmental disturbances and are at a competitive disadvantage in resource-limited soils (26, 27). In subtropical forest ecosystems, the biological release of available P from soil recalcitrant Po is regulated not only by phosphatases but also by other hydrolytic and oxidative enzymes (6, 28–30). Thus, to facilitate Po mineralization, microorganisms may have evolved special survival strategies through division of labor and cross-feeding with diverse communities. For example, Chen et al. (31) found that keystone fungal taxa modulate P availability by interacting with Po-mineralizing fungal taxa under long-term P fertilization. This cooperation between Po-mineralizing taxa may improve soil P availability, but a substantial interaction of Po-mineralizing microorganisms with the rest of the soil microbial community is needed to determine P availability in forest ecosystems.

In this study, we investigated the associations between Po-mineralization capacity and the soil microbial community. We collected soil samples from evergreen broadleaf secondary forests, mixed forests planted mainly with Chinese fir (*Cunninghamia lanceolata*), and pure Chinese fir forests in southeastern China. China accounts for 71% of the total subtropical forest area globally, and these subtropical forests in China are mainly composed of Chinese fir forests and evergreen broadleaf secondary forests (10, 32). We characterized the soil P fractions, Po-mineralization ability, and associated microbes. The effects of microbial interactions on Po mineralization were further investigated by examining the status of Po-mineralizing taxa in the microbial community and by performing *in vitro* bioassays. We hypothesized that (i) soil Po mineralization is associated with the soil microbial community in different forest types and (ii) interactions of Po-mineralizing microbes with the rest of the soil microbial community determine Po-mineralization ability (31, 33).

## MATERIALS AND METHODS

### Study site and field sampling

The study site is located at Xiayang Forestry Station (27°17′N, 118°01′E), Nanping City, southeastern China. The region is characterized by a montane temperate climate. The mean annual temperature and precipitation are 20°C and 1,669 mm, respectively. In 1990, three forest types were established: (i) pure Chinese fir forest (PCF), (ii) mixed planting with Chinese fir and other broadleaf species forest (MCF), and (iii) secondary natural evergreen broadleaf forest (SNF). In the PCF, only Chinese fir with a density of 900 trees ha^−1^ was planted. In the MCF, Chinese fir and *Schima superba* were interplanted simultaneously at a ratio of 7:3 and a density of 900 trees ha^−1^. In the SNF, multiple tree species were allowed to develop through natural competition without any anthropogenic disturbance after deforestation. The dominant species, density, slope, aspect, and elevation of the forest types are provided in Table S1. For each type of forest, three replicate blocks with an area of ~0.5 ha were randomly distributed at a boundary distance of 50 m. No thinning, weed control, or fertilization were performed for these blocks in the past 20 years, and all trees were well grown and free of pests and diseases. The soil in the area developed from acidic loamy clay red soil and is a Ferralic Cambisol.

In December 2021, after removing surface litter, 15 soil cores (0–20 cm) in each forest block were collected following an “S” sampling pattern and were evenly mixed to form a composite sample. Three replicates of soil samples were thus employed for each forest type. These fresh samples were immediately transported to the laboratory in ice incubators. Then, the fresh samples were passed through a 2-mm sieve to remove visible residues, homogenized, and divided into two portions. One portion was stored at 4°C and used for the analysis of soil P fractions and corresponding physicochemical properties, cultivation experiments, and enzymatic activity detection; the other portion was stored at −80°C and used for DNA extraction.

### Soil P fractions

Soil P fractions were determined using a sequential extraction procedure developed by Hedley et al. (34) and modified by Tiessen and Moir (35) and Gao et al. (36). In brief, soil samples (2.0 g) were sequentially extracted as follows: (i) Resin-Pi was extracted using 1.0 g of anion-exchange resin eluted with 0.5 M HCl; (ii) NaHCO_3_-P was extracted with 0.5 M NaHCO_3_ (pH 8.5); (iii) NaOH-P was extracted with 0.1 M NaOH; (iv) HCl-P was extracted with 1.0 M HCl; and (v) residual P was extracted with H_2_SO_4_ and 30% H_2_O_2_. The concentrations of inorganic NaHCO_3_-P (NaHCO_3_-Pi) and inorganic NaOH-P (NaOH-Pi) were determined by the phosphomolybdic blue colorimetric method. Total P (TP) in NaHCO_3_ and NaOH extracts was determined after digestion by K_2_S_2_O_8_. Then, organic NaHCO_3_-P (NaHCO_3_-Po) and organic NaOH-P (NaOH-Po) were calculated as the difference between TP and Pi. Based on the turnover rate and solubility of different P fractions (37, 38), the resin-Pi and NaHCO_3_-P fractions represent easily available P (labile P), the NaOH-P fraction represents moderately available P (moderately labile P), and the HCl-Pi and residual P fractions represent unavailable P (stable P).

### Soil physicochemical and biological properties

Soil pH was determined with a pH meter in a 1:2.5 (wt/vol) soil-deionized CO_2_-free water suspension. Soil organic carbon (SOC) and total nitrogen (TN) contents were measured using the K_2_Cr_2_O_7_ external heating oxidation method and the semi-Kjeldahl method, respectively (39). Soil nitrate nitrogen (NO_3_^−^-N) and ammonium nitrogen (NH_4_^+^-N) were extracted with 2 M KCl and determined spectrophotometrically (Lambda 950, PerkinElmer, Inc.). Soil total potassium (TK) and available potassium (AK) were measured by using a flame spectrophotometer (FP6450, Nanbei Instrument Equipment Co., Ltd.). Soil microbial biomass carbon (MBC), nitrogen (MBN), and phosphorus (MBP) were determined in fresh samples by using the chloroform fumigation–extraction method (40, 41). ACP activity in soil was determined by incubating it with p-nitrophenyl phosphate (p-NPP) as a substrate at pH 6.0 for 1 h (42).

### Identification of Po-mineralization capacity

Modified Pikovskaya’s medium [1 L of medium containing 10 g of glucose, 0.5 g of (NH_4_)_2_SO_4_, 0.5 g of lecithin, 0.3 g of KCl, 0.03 g of MnSO_4_ ·H_2_O, 0.03 g of FeSO_4_7·H_2_O, 0.3 g of NaCl, and 0.03 g of MgSO_4_·7H_2_O] is widely employed to identify Po-mineralizing microorganisms (31, 43). In this study, we adjusted the pH of the modified Pikovskaya’s medium to 5.5 to match the soil pH (5.12–5.53) of the three forest types. The parent soil was incubated at 30°C for 5 days, and then 5 g of parent soil was diluted with 45 mL of sterilized water and oscillated at 180 rpm for 60 min to create parent soil suspension. One milliliter of the parent soil suspension was transferred to 120 mL of modified Pikovskaya’s medium and incubated at 30°C on a rotating shaker at 160 rpm for 120 h. The negative control was prepared in the same manner, except that 1 mL of sterile water was added instead of the parent soil suspension. Cultures were prepared in triplicate for each forest type. During cultivation, 10 mL of medium was collected every 12 h under sterile conditions. The collected medium was centrifuged at 5,000 rpm for 5 min, and the supernatant was used to measure the Po concentration, alkaline phosphatase (ALP) and ACP activities, and pH. The ACP and ALP activities in the supernatant were determined by incubation with p-NPP as a substrate at pH 6.0 and 8.0 for 1 h, respectively (42).

### DNA extraction and Illumina sequencing

Genomic DNA was extracted from the soil and centrifugal precipitated by using the MP FastDNA spin kit (MP Biomedicals, Solon, OH, USA) according to the manufacturer’s instructions. The quality of the extracted DNA was confirmed by using a NanoDrop 2000 spectrophotometer (Thermo Scientific, Wilmington, USA). The bacterial 16S rRNA and fungal ITS region were amplified using the primer pairs 515F/806R and ITS1F/ITS2, respectively (44). The resulting sequences were filtered to remove primer sequences, chimeras, and low-quality reads with a quality score below 30 by using QIIME2 (45). Gene sequencing was performed on the Illumina HiSeq2500 platform (Illumina, San Diego, CA, USA) by Shanghai Personal Biotechnology Co., Ltd. (Shanghai, China). The DADA2 plugin was employed for quality control, and amplicon sequence variants (ASVs) were identified (46). The ASVs were taxonomically assigned using the SILVA database (http://www.arb-silva.de, version:138.1) for bacteria and the UNITE database (release 7.2, http://unite.ut.ee/index.php) for fungi.

### Po-mineralizing microorganism identification

The modified Pikovskaya’s medium has been widely and successfully used to identify Po-mineralizing microorganisms (31, 43, 47). Generally, one certain time point during the culture process was employed in the previous publications (31). To improve the accuracy of the Po-mineralizing microorganism identification, we tracked the changes in microbial composition with an interval of 24 h during the culture experiment. In the modified Pikovskaya’s medium, although some microorganisms may take advantage of P mineralized by Po-mineralizing microorganisms for growth, Po-mineralizing microorganisms have a competitive advantage and can grow quickly. Since we observed a significant increase in ACP activity after 48 h and observed maximum ACP activity at 84 h, the centrifugal precipitates at 24, 48, and 84 h were collected for DNA extraction and to identify Po-mineralizing microorganisms. Po-mineralizing microorganisms were identified at the ASV level according to the following criteria: (i) increase in abundance with culture time, (ii) appearance in at least two replicate samples, and (iii) total reads > 30.

### Dilution assays of the effect of microbial interactions on Po-mineralization capacity

Soil suspension dilutions were performed to examine the effect of weakened microbial interactions on Po mineralization, as the loss of soil microbial diversity due to dilution leads to simple interactions (48, 49). The parent soil suspension was serially diluted to create a gradient of undiluted (D1) to diluted 10^−3^ (D3) and 10^−5^ (D5) suspensions. After dilution, the three soil suspensions (D1, D2, and D3) were transferred to Pikovskaya’s medium in the same manner as described in the section “Identification of Po-mineralization capacity”, respectively. The dilutions were incubated at 30°C on a rotating shaker at 160 rpm for 120 h. ACP activity was measured every 24 h, and the Po content in the medium was analyzed at the end of the culture (120 h).

### Statistical analysis

Duncan’s *post hoc* multiple comparison tests were implemented to assess the differences in soil and microbial community properties between different forest types and cultivation times in Welch’s one-way analysis of variance by using the “agricolae” package. All differences were considered significant at *P* < 0.05. Microbial alpha diversity indices (Shannon and richness) were evaluated by using the “vegan” package. Microbial beta diversity was quantified by Bray–Curtis dissimilarity-based principal coordinate analysis (PCoA) in the “vegan” package. The differences between the microbial communities were identified with permutational multivariate analysis of variance (PERMANOVA) through the 999 permutations by using the Adonis function in the “vegan” package. Linear discriminant analysis effect size was used to identify the most discriminant soil ASVs between different forest types (50, 51).

Co-occurrence networks of the cultured samples and soil samples were constructed based on ASV relative abundance to gain insights into the putative ecological interactions and structure of the microbial communities. Spearman’s correlation (| *r* | > 0.8; *P* < 0.05)-based networks of bacterial and fungal communities were constructed by using the “Hmisc” package. The *P* values were adjusted using Benjamini and Hochberg’s false discovery rate (52). Topological properties, including modularity, average degree, and number of edges, were used to assess microbial network characteristics. Furthermore, the topological properties of the within-module connectivity (Zi) and among-module connectivity (Pi) of each node in the co-occurrence networks of the cultured samples were determined (53). According to the Zi and Pi values, connectors (Zi < 2.5 and Pi > 0.62), module hubs (Zi > 2.5 and Pi < 0.62), network hubs (Zi > 2.5 and Pi > 0.62), and peripherals (Zi < 2.5 and Pi < 0.62) were identified (54). The networks were visualized in Gephi software (v. 0.9.2). All statistical analyses were performed in R (v. 4.0.2).

## RESULTS

### Differences in soil P availability and stoichiometry among forest types

**Fig 1:**
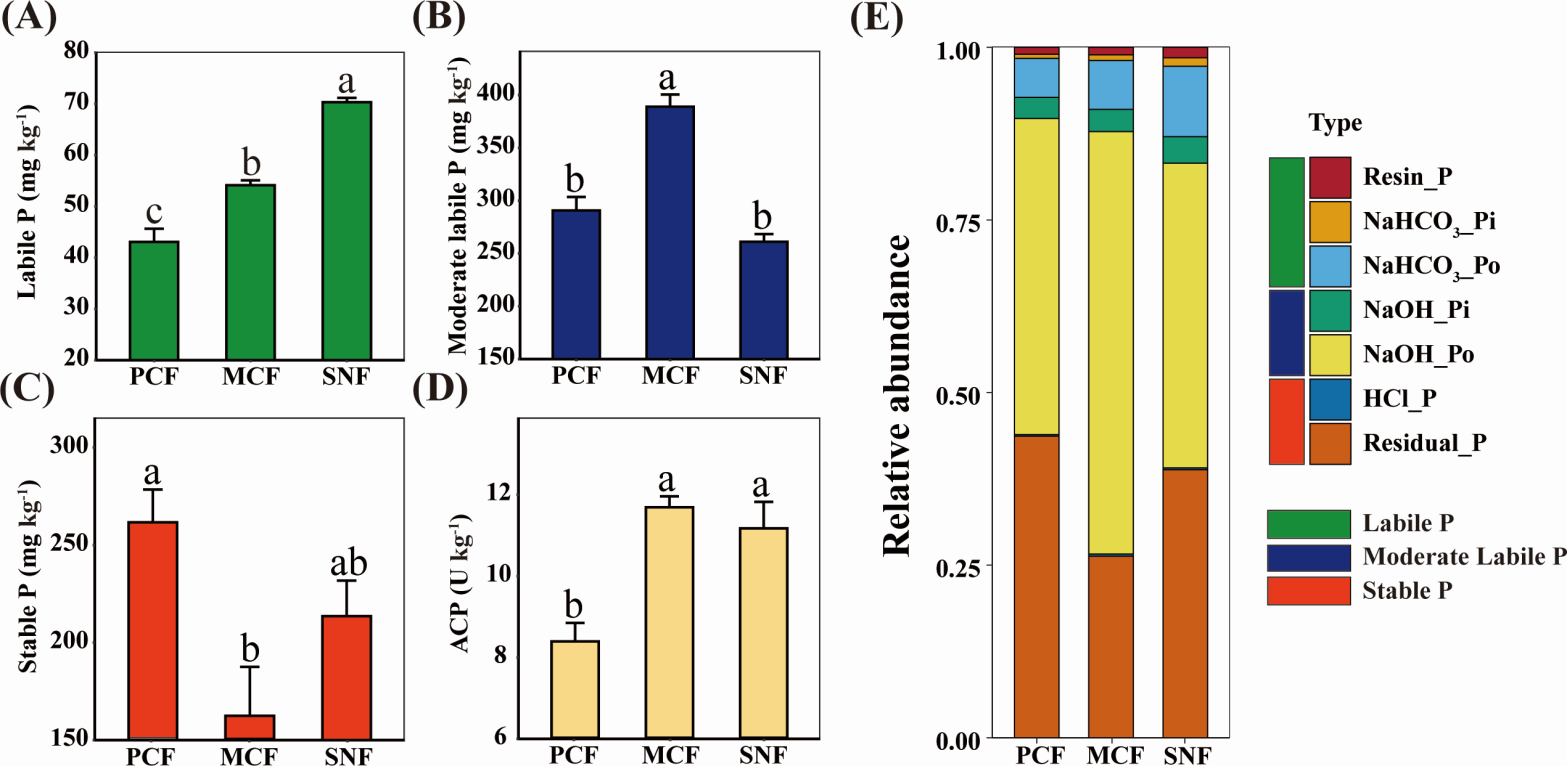
Concentrations of different phosphorus (P) fractions and acid phosphatase activity in the three forests. (A) Soil labile P in the three forests. (B) Soil moderate labile P in the three forests. (C) Soil stable P in the three forests. (D) Soil ACP in the three forests. (E) Proportion of different P fractions in the three forest types. PCF: pure Chinese fir forest; MCF: mixed Chinese fir forest; SNF: secondary natural forest. The values are means ± SEs (*n* = 3). Different letters indicate statistical significance at *P* = 0.05.

Forest type significantly influenced soil P fractions and availability but not soil TP. The patterns of soil P fractions were similar across the three forest types, but the contents of Resin-P, NaHCO_3_-Pi, and NaHCO_3_-Po were highest in SNF, followed by MCF and PCF, whereas NaOH-Po content was highest in MCF (Fig. S1). Labile P (Resin-P and NaHCO_3_-P) accounted for 7.24%–12.90% of TP in the forest soils. The labile P content was 70.31 mg kg^−1^ in SNF, 22.6% and 38.7% higher than in MCF and PCF, respectively (Fig. 1). Moderately labile P content did not differ significantly between SNF and PCF, whereas stable P content was higher in PCF (261.63 mg kg^−1^) than in the other forest types. Consistent with the variation in labile P among the three forest types, soil ACP activity was highest in SNF, followed by MCF and PCF. In addition, SOC, TN, TK, NH_4_^+^-N, MBC, MBN, and MBP contents were significantly higher in SNF than in MCF and PCF (Table 1).

**TABLE 1:** Soil physicochemical properties in the three forests^*a*^.

	pH	TN (g kg^−1^)	SOC (g kg^−1^)	TP (mg kg^−1^)	TK (g kg^−1^)	AK (mg kg^−1^)	NO_3_^−^-N (mg kg^−1^)	NH_4_^+^-N (mg kg^−1^)	MBC (mg kg^−1^)	MBN (mg kg^−1^)	MBP (mg kg^−1^)
PCF	5.12 ± 0.21b	1.26 ± 0.09c	41.14 ± 1.01b	595.25 ± 9.25a	61.67 ± 2.02b	7.30 ± 1.71b	2.58 ± 0.55a	9.01 ± 0.72c	1,067.53 ± 27.63b	56.62 ± 4.47b	6.47 ± 0.31b
MCF	5.53 ± 0.04a	1.41 ± 0.03b	42.79 ± 1.37b	605.62 ± 48.74a	63.00 ± 6.56b	24.17 ± 1.07a	2.10 ± 0.12a	21.94 ± 1.54b	1,767.99 ± 90.76a	159.23 ± 18.87a	6.68 ± 0.83b
SNF	5.53 ± 0.22a	2.11 ± 0.10a	62.70 ± 1.32a	544.90 ± 23.51a	77.83 ± 7.85a	27.93 ± 6.18a	3.92 ± 1.68a	38.93 ± 3.80a	1,510.84 ± 326.82a	129.81 ± 30.44a	12.03 ± 0.99a

^*a*^ TN: total nitrogen; SOC: soil organic carbon; TP: total phosphorus; TK: total potassium; AK: available potassium; NO_3_^−^-N: nitrate nitrogen; NH_4_^+^-N: ammonium nitrogen; MBC: microbial biomass carbon; MBN: microbial biomass nitrogen; MBP: microbial biomass phosphorus; PCF: pure Chinese fir forest; MCF: mixed Chinese fir forest; SNF: secondary natural forest. The values are means ± SEs (*n* = 3). Different letters within each column indicate statistical significance at *P* = 0.05.

### Dynamics of soil Po-mineralization capacity in three forests

**Fig 2:**
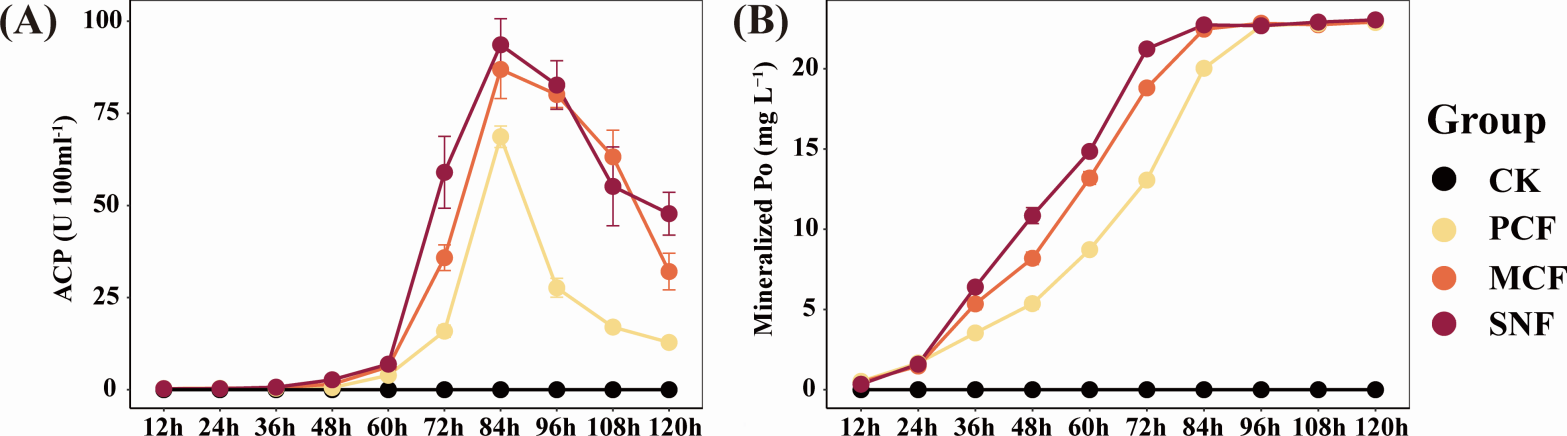
Dynamics of organic phosphorus mineralization in the three forest types during culture. (**A**) Acid phosphatase activity. (**B**) Mineralized organic phosphorus content. PCF: pure Chinese fir forest; MCF: mixed Chinese fir forest; SNF: secondary natural forest; Po: organic phosphorus. Error bars denote the standard error of the mean (*n* = 3).

We determined the Po-mineralization capacity of the forest soil by using lecithin as the sole source of P in the culture medium. The pH of the medium dropped sharply after 24 h (Fig. S4), which indicated that mineralization of lecithin by soil microbes was occurring. The Po mineralization capacity remained synchronous with the decrease in pH and peaked at 84 h. The efficiency of Po mineralization was higher in SNF than in MCF and PCF, and most of the Po was exhausted before 84 h (Fig. 2B). We tracked ACP activity at 12-h intervals, which revealed that ACP activity increased rapidly after 48 h and reached a maximum at 84 h (Fig. 2A). Before reaching the maximum, ACP activity was highest in SNF, followed by MCF and PCF. ALP activity was not detected in the medium during the culture experiment (data not shown), suggesting that ACP released by forest soil microbes was responsible for Po mineralization.

### Fungal and bacterial communities associated with Po mineralization

**Fig 3:**
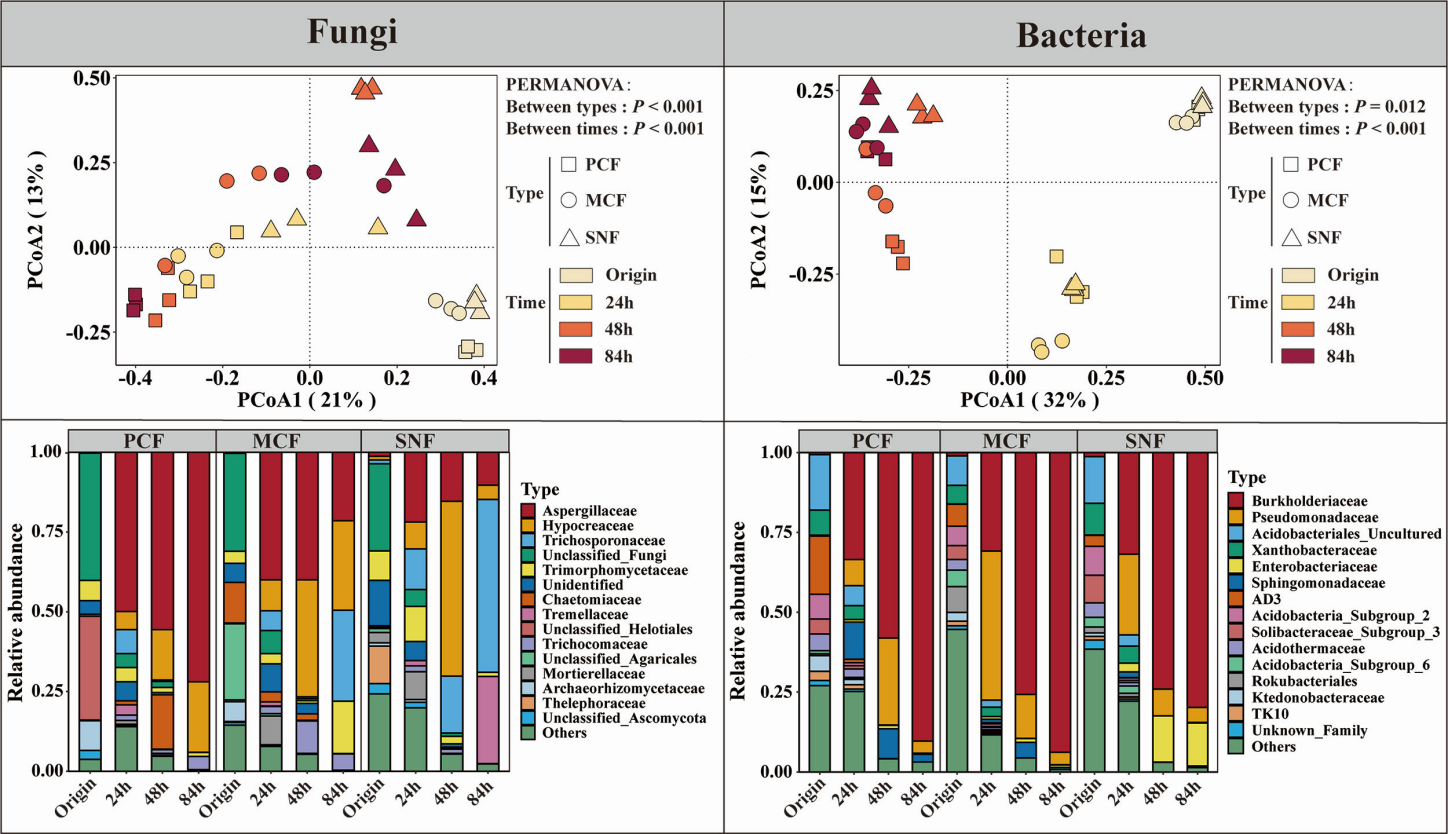
Principal coordinate analysis of soil microbial communities (based on Bray‒Curtis distances) and soil microbial community composition in the three forest types during culture. The stacked bar chart shows the relative abundances of the top 15 microbial families. PERMANOVA: permutational multivariate analysis of variance (999 permutations). PCF: pure Chinese fir forest; MCF: mixed Chinese fir forest; SNF: secondary natural forest.

The PCoA revealed a separation of the bacterial and fungal communities not only between different culture times but also between forest types (Fig. 3). The separation of the fungal community between forest types increased with increasing culture time, and PCF showed the greatest separation from MCF and SNF at 84 h. Moreover, the separation of the fungal community between culture times was greater than the separation between forest types. For the bacterial community, the separation increased with increasing culture time, and the separation between culture times was greater than the separation between forest types.

With increasing culture time, community succession occurred as a consequence of the decreases in fungal and bacterial community diversity (Fig. 3; Fig. S3). The differences in fungal community composition between forest types increased during culture, while bacterial community composition showed the opposite trend. Specifically, at 84 h, Aspergillaceae contributed 71.9% of the total abundance of PCF, Hypocreaceae (28.1%) and Trichosporonaceae (28.5%) were the predominant families in MCF, and Hypocreaceae (54.1%) and Tremellaceae (27.3%) were the most abundant families in SNF. By contrast, the bacterial communities of the three forest types at 84 h were similar, and Burkholderiaceae was the predominant family, accounting for more than 80% of the total bacterial abundance for all forest types.

**Fig 4:**
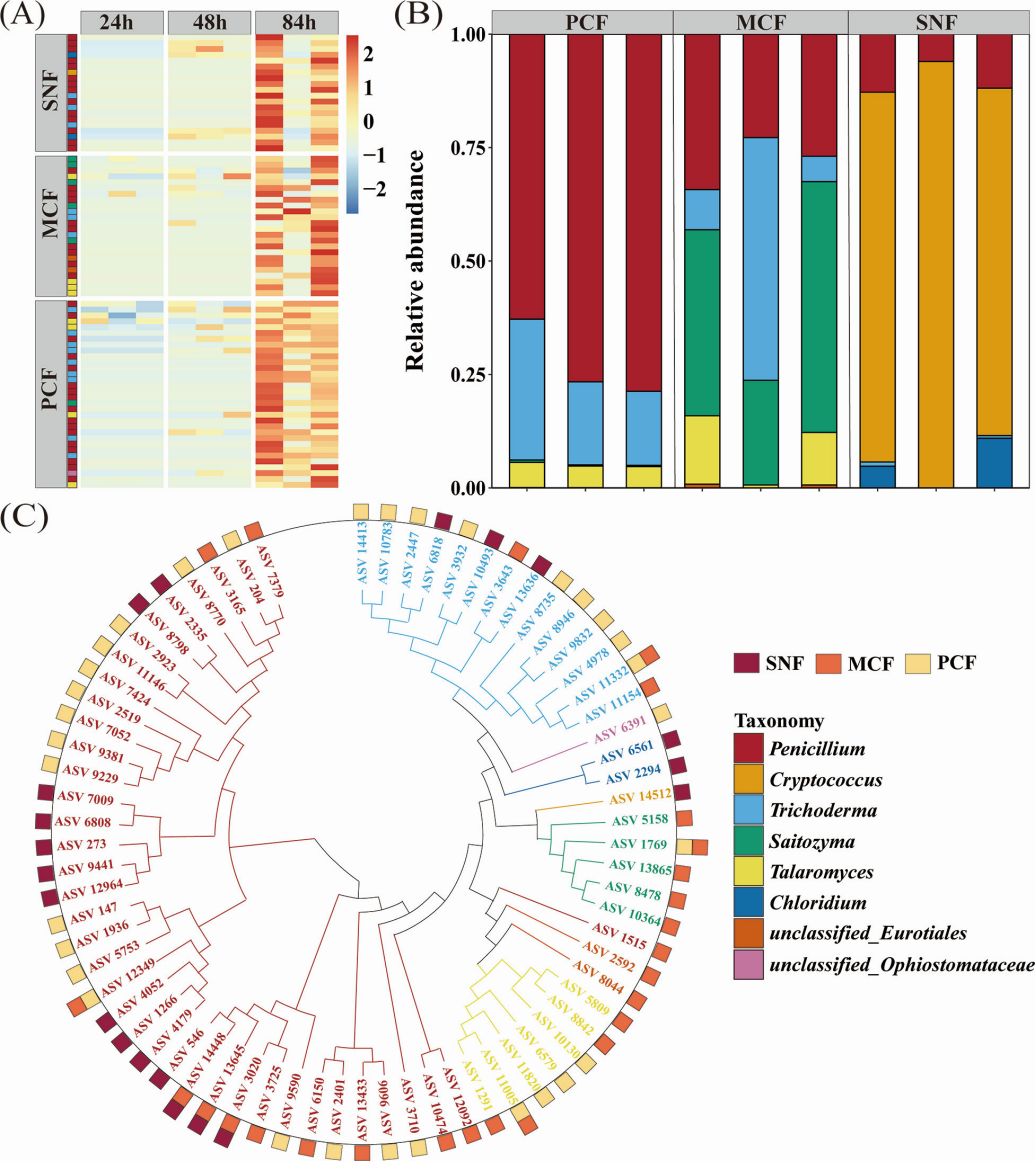
Identification of organic phosphorus-mineralizing fungi. (**A**) Changes in the relative abundances of organic phosphorus-mineralizing fungi at the ASV level. (**B**) Proportions of organic phosphate-mineralizing fungi at the genus level in the different forest types. (**C**) Phylogenetic relationships among the organic phosphorus-mineralizing fungi. PCF: pure Chinese fir forest; MCF: mixed Chinese fir forest; SNF: secondary natural forest.

Since ACP activity increased from 24 to 84 h, we identified Po-mineralizing microorganisms at the ASV level whose abundance covaried with ACP activity. A total of 86 fungal ASVs and 114 bacterial ASVs were enriched at 84 h (Fig. 4; Fig. S5). The Po-mineralizing fungi in PCF (Pmin-PCF) mainly comprised *Penicillium* (72.7%). *Trichoderma* (22.6%) and *Saitozyma* (39.8%) were the predominant Po-mineralizing fungi in MCF (Pmin-MCF). By contrast, *Cryptococcus* (84.0%) was the most abundant Po-mineralizing fungal taxon in SNF (Pmin-SNF). In all forest types, *Burkholderia_Caballeronia_Paraburkholderia* was the predominant Po-mineralizing bacterial taxon and contributed 78%–99% of the total abundance of Po-mineralizing bacteria. The phylogenetic tree of Po-mineralizing microorganisms further indicated that the Po-mineralizing fungi were phylogenetically diverse and rarely overlapped between forest types. By contrast, the phylogenetic diversity of Po-mineralizing bacteria was limited, and the same species were often detected in different forest types. Moreover, Po-mineralizing microorganisms were present in low abundance in the soil microbial community and accounted for 1.5% and less than 0.1% of soil fungal and bacterial abundance, respectively.

### Complex interactions of the soil microbial community promote Po mineralization

**Fig 5:**
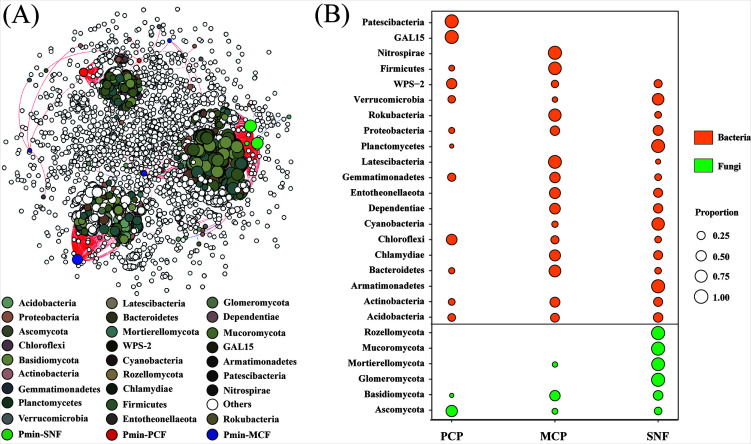
Associations between organic phosphorus-mineralizing microorganisms and the rest of the soil microorganisms. (**A**) The node size indicates the degree of the node, red lines indicate positive correlations and green lines indicate negative correlations; Pmin-SNF, Pmin-MCF, and Pmin-PCF indicate organic phosphorus-mineralizing microorganisms in SNF, MCF, and PCF, respectively. Other colored nodes represent the rest of the soil microorganisms that are significantly associated with soil organic phosphorus-mineralizing microorganisms (**B**) The soil microorganisms (phyla level) that are significantly associated with soil organic phosphorus-mineralizing microorganisms in the three forest types. Each node represents a microorganism, and the size of the node represents the proportion, and the sum of the proportions in each row is one. PCF: pure Chinese fir forest; MCF: mixed Chinese fir forest; SNF: secondary natural forest.

The co-occurrence network of the microbial community accompanying Po mineralization had a more than 10-fold higher degree of interactions with Po-mineralizing fungi than with Po-mineralizing bacteria (Table S2). Moreover, Pmin-SNF had stronger positive interactions with more diverse soil microorganisms than Pmin-MCF and Pmin-PCF (Fig. 5) and acted as a network hub (Fig. S6). Among the soil microorganisms that were significantly correlated with Po-mineralizing fungi, fungi accounted for more than 70% of the microbial abundance and were most enriched in SNF (Fig. 5B), suggesting widespread cooperation of soil fungi with Po-mineralizing fungi.

**Fig 6:**
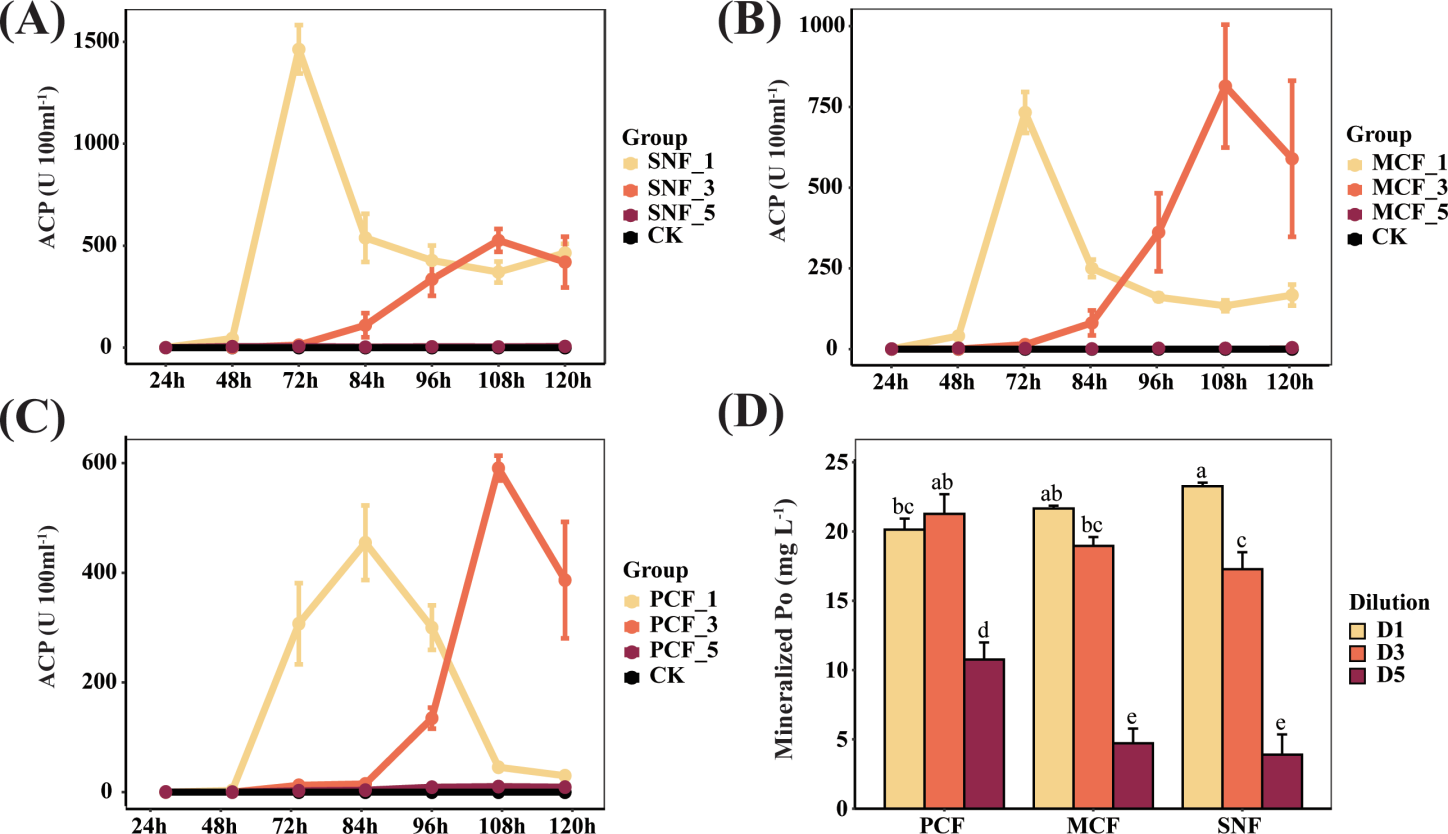
The effect of dilution on the dynamics of acid phosphatase activity and organic phosphorus content in the medium of SNF, MCF, and PCF, respectively. The numbers 1, 3, and 5 following the abbreviation of the forest type denote no dilution and dilution ratios of 1,000 or 100.000, respectively. PCF: pure Chinese fir forest; MCF: mixed Chinese fir forest; SNF: secondary natural forest. The error bars denote the standard error of the mean (*n* = 3). Different letters indicate significant differences among forests at a given dilution at *P* = 0.05.

To examine the effect of interaction intensity on Po-mineralization capacity, we diluted the parent soil suspensions to create varying degrees of species loss. Dilution not only reduced ACP activity but also delayed the time for ACP activity to reach its maximum (Fig. 6A through C). At moderate dilution (D3), the ACP activity of MCF and PCF reached a maximum at 108 h, while the ACP activity of SNF decreased by 303.9% and was unrecoverable. At the highest dilution (D5), no increase in ACP activity was observed during the entire culture experiment for all three forest types (Fig. 6; Fig. S8), which demonstrated that microbial interactions that were not involved in Po mineralization had no Po-mineralization activity. Moreover, moderate soil dilution significantly decreased the mineralized Po content in SNF but did not affect the mineralized Po content in MCF and PCF (Fig. 6D).

## DISCUSSION

Po mineralization steadily produces soil available P to support forest primary productivity (55–57). Diverse soil microorganisms account for up to 90% of Po mineralization in forest ecosystems (7, 58), but few studies have focused on the effects of Po-mineralizing microbial composition and their interactions on forest soil Po mineralization at the community level. In the present study, we found that soil P availability in three forests was associated with higher Po-mineralization capacity of the soil microbial community. Our examination of the effects of the soil microbial community on Po mineralization using multiple approaches indicated key roles of cooperation between Po-mineralizing fungi and the remainder of the soil microbial community in regulating P availability.

At the study site, labile P accounted for 7.24%–12.90% of soil TP, a lower range than those reported in other studies of subtropical forests (9, 10). This low labile P suggests that the productivity of subtropical forests is constrained by soil P availability (38). However, we found that forest type strongly influenced soil P availability; namely, soil labile P content was higher in SNF than in MCF and PCF, consistent with previous studies in subtropical regions (3, 9) and temperate regions (59). The high soil labile P in SNF may be related to the high availability of soil nutrients (SOC, TN, NH_4_^+^-N, AK) to promote microbial metabolism, since microbial carbon acquisition releases P as a byproduct (60–63). To evaluate the Po-mineralization characteristics of the soil microbial community in different forest types, we cultured soil suspensions with lecithin to avoid the influence of soil properties on the Po-mineralization process. We found that soil Po mineralization was more efficient in SNF than in MCF and PCF due to high ACP secretion, which contributed to high Po mineralization in media inoculated with SNF soil suspension (5, 9, 21). Our results emphasize the importance of microbial Po mineralization for soil P availability in the absence of externally available P (64).

Microbial community dynamics were tracked from the onset to the peak of ACP activity (24–84 h). We found that both the diversity and the abundance of the bacterial community decreased over time (Fig. 3; Fig. S3), indicating a competitive advantage of certain bacteria in mineralizing Po. Contrary to the homogenization of the bacterial community during culture, the fungal community clearly differed among the three forest types, implying differentiation of the fungal community involved in Po mineralization. Because ACPs are encoded by a variety of genes, such as acpA, phoC, and napA (65), ACP-secreting capacity may differ between different Po-mineralizing microorganisms, resulting in different Po-mineralization abilities. We identified Po-mineralizing taxa by screening for microorganisms whose abundances covaried with ACP activity during culture. We found that diverse phylogenetic lineages of Po-mineralizing fungi were enriched in the cultures of soil from the different forest types (Fig. 4). Specifically, the most abundant Po-mineralizing fungal taxon in SNF was *Cryptococcus*, whereas *Penicillium* and *Trichoderma* were the predominant Po-mineralizing fungi in MCF and PCF. *Penicillium* and *Trichoderma* were previously reported to have P-mobilization ability in a variety of soil environments (33, 66), and *Cryptococcus* has been linked to increases in enzymatic activities related to litter degradation and P cycling (67). By contrast, the Po-mineralizing bacteria of all three forest types were dominated by *Burkholderia_Caballeronia_Paraburkholderia* (Fig. S5), which has been reported to have potential P-solubilization capacity (68).

Soil Po is recalcitrant and can only be degraded with the participation of phosphatases, which are mainly secreted by Po-mineralizing microorganisms (21). The superior P-acquisition ability of Po-mineralizing microorganisms may give them an absolute advantage in niche occupation, resulting in increased abundance at the expense of other microorganisms. Nonetheless, Po-mineralizing microorganisms are present in low abundance in various soils (7, 31). This suggests that the realization (or at least enhancement) of Po mineralization driven by Po-mineralizing microorganisms may depend on cooperation among microorganisms with multiple metabolic functions (29), as the adaptability of soil microorganisms to the environment depends on interactions (69, 70). Network construction revealed intense positive correlations between Po-mineralizing fungi and the rest of the soil microbial community in soils with strong Po-mineralization capacity. The networks of the cultured samples also indicated that microbial interactions were greater in SNF than in PCF (Fig. S6 and S7), further confirming the positive relationship between Po-mineralization ability and microbial interactions. Cooperative relationships such as cross-feeding between microorganisms allow the use of a wider range of resources to survive in resource-limited soils (63, 71). In forest ecosystems, soil P pools are mainly bound in recalcitrant litter, and the mineralization of Po requires the participation of a wide range of extracellular enzymes (22, 27). Soil microbes that do not secrete phosphatases can still promote the transformation of refractory Po by secreting oxidases, hydrolases, and cellulases (22, 30, 72), thereby increasing substrate availability for phosphatases. Consequently, cooperative relationships between Po-mineralizing fungi and a variety of microorganisms play key roles in promoting Po mineralization (22, 73).

**Fig 7:**
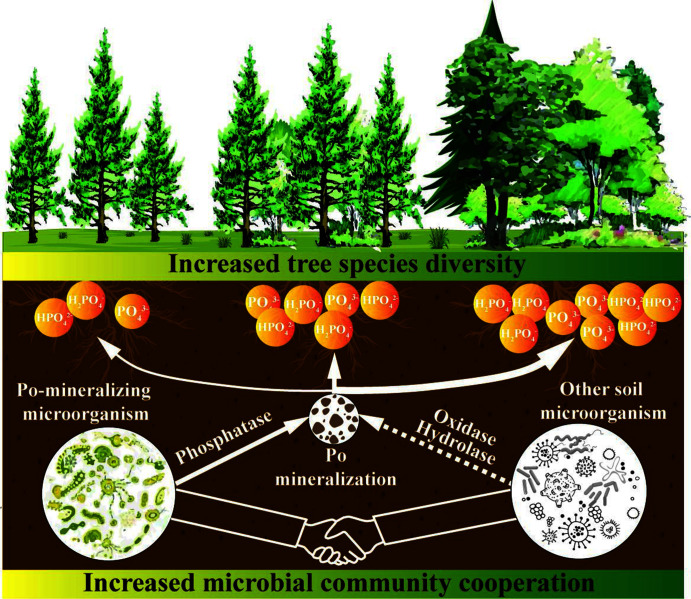
Relationship between soil microbial community cooperation and soil phosphorus availability. Po: organic phosphorus.

However, correlation coefficients cannot be uncritically considered as ecological interactions as the “interactions” in the network are only mathematical relationships (74, 75). Therefore, whether the positive correlation in the network represents ecological cooperation needs to be further investigated (76). We further verified the effect of microbial interactions on Po-mineralization capacity by performing gradient dilutions of the parent soil suspensions. Dilution may result in the loss of soil microbes and consequently weaken the interactions between Po-mineralizing microorganisms and other microorganisms (48, 49). We found that moderate dilution delayed ACP production, further confirming that microbial interactions are one of the main factors regulating Po mineralization. Most importantly, in SNF, where the Po-mineralizing microorganisms had the strongest interactions with other microorganisms, moderate dilution (D3) resulted in unrecoverable ACP activity, which indicated that microbial interactions play an important and irreplaceable role in Po mineralization. Additionally, we did not directly measure the effect of dilution on microbial diversity and abundance; nevertheless, Po was consumed profoundly at the end of cultivation (120 h) regardless of the dilution ratio, implying that microbial interactions have a greater impact on the mineralization of Po than microbial abundance. Indeed, the mineralized Po content was significantly lower in the D3 dilution of SNF than in the D1 dilution, whereas mineralized Po content did not differ significantly among the dilutions of MCF and PCF. However, it would be more convincing to directly measure the reduction in microbial community diversity caused by dilution and to model the transformation of P between biomass and medium in future studies. In summary, our results highlight the importance of microbial interactions in improving P availability (Fig. 7) and suggest that more attention should be given to microbial interactions in future studies of measures to reduce P stress in plantations.

### Conclusions

By combining soil samples and targeted laboratory incubations, this study clarified the mechanism of microbial-involved soil Po mineralization in forest ecosystems of subtropical regions. We found that the higher Po-mineralization ability of SNF soil contributed to the high labile P content of SNF compared to MCF and PCF. Although Po-mineralizing fungi differed among the forest types, interactions between Po-mineralizing fungi and other soil microorganisms were beneficial for improving Po-mineralizing capacity in all forest types. The weakening of these interactions led to a decline in Po-mineralization capacity, indicating the importance of protecting microbial diversity in forest soils. Our results strengthen our understanding of how microorganisms regulate Po mineralization in forest soils.

## Data Availability

The sequence data were deposited in the National Center for Biotechnology Information (NCBI) Sequence Read Archive (SRA) database (accession no. PRJNA916980 for fungi and PRJNA917026 for bacteria).

## SUPPLEMENTAL MATERIAL

Supplementary tables and figures spectrum.01355-23-s0001.docx This file contains 2 supplementary tables and 8 supplementary figures.

ASM does not own the copyrights to Supplemental Material that may be linked to, or accessed through, an article. The authors have granted ASM a non-exclusive, world-wide license to publish the Supplemental Material files. Please contact the corresponding author directly for reuse.
