# Supplementary material for: Microbial community interactions determine the mineralization of soil organic phosphorus in subtropical forest ecosystems
Source: Microbiol Spectr. 2024 Feb 9;12(3):e01355-23. doi: 10.1128/spectrum.01355-23 (PMC10913379; doi:10.1128/spectrum.01355-23)

**Table S1.** Basic information on the forests.

| **Type** | **Replicates** | **Dominant species** | **Slope** | **Aspect** | **Canopy density** | **Elevation (m)** |
| --- | --- | --- | --- | --- | --- | --- |
| Secondary natural forests | Replicates 1 | *Schima superba* (20%) | 21° | West | 0.6 | 470 |
|  |  | *Lithocarpus glaber* (15%) |  |  |  |  |
|  |  | *Zelkova serrata* (14%) |  |  |  |  |
|  |  | *Castanea henryi* (14%) |  |  |  |  |
|  |  | *Cyclobalanopsis glauca* (11%) |  |  |  |  |
|  |  | *Sassafras tzumu* (8%) |  |  |  |  |
|  |  | *Choerospondias axillaris* (7%) |  |  |  |  |
|  |  | Others (11%) |  |  |  |  |
|  | Replicates 2 | *Schima superba* (19%) | 23° | Southwest | 0.7 | 450 |
|  |  | *Lithocarpus glaber* (17%) |  |  |  |  |
|  |  | *Zelkova serrata* (13%) |  |  |  |  |
|  |  | *Castanea henryi* (13%) |  |  |  |  |
|  |  | *Cyclobalanopsis glauca* (11%) |  |  |  |  |
|  |  | *Phoebe bournei* (9%) |  |  |  |  |
|  |  | *Michelia chapensis Dandy* (8%) |  |  |  |  |
|  |  | Others (10%) |  |  |  |  |
|  | Replicates 3 | *Schima superba* (21%) | 19° | West | 0.6 | 460 |
|  |  | *Lithocarpus glaber* (20%) |  |  |  |  |
|  |  | *Zelkova serrata* (12%) |  |  |  |  |
|  |  | *Castanea henryi* (11%) |  |  |  |  |
|  |  | *Manglietia fordiana Oliv* (8%) |  |  |  |  |
|  |  | *Delonix regia* (8%) |  |  |  |  |
|  |  | *Erythrina variegata* (7%) |  |  |  |  |
|  |  | Others (13%) |  |  |  |  |
| Mixed Chinese fir forests | Replicates 1 | *Cunninghamia lanceolata* (67%) | 24° | Northwest | 0.6 | 260 |
|  |  | *Schima superba* (19%) |  |  |  |  |
|  |  | *Sassafras tzumu* (5%) |  |  |  |  |
|  |  | *Hamamelidaceae*(4%) |  |  |  |  |
|  |  | Others (5%) |  |  |  |  |
|  | Replicates 2 | *Cunninghamia lanceolata* (63%) | 27° | Northwest | 0.6 | 250 |
|  |  | *Schima superba* (21%) |  |  |  |  |
|  |  | *Erythrina variegata* (6%) |  |  |  |  |
|  |  | *Phoebe bournei*(5%) |  |  |  |  |
|  |  | Others (5%) |  |  |  |  |
|  | Replicates 3 | *Cunninghamia lanceolata* (65%) | 28° | Northwest | 0.6 | 270 |
|  |  | *Schima superba* (20%) |  |  |  |  |
|  |  | *Ficus hirta Vahl* (7%) |  |  |  |  |
|  |  | *Hamamelidaceae*(4%) |  |  |  |  |
|  |  | Others (4%) |  |  |  |  |
| Pure Chinese fir forests | Replicates 1 | *Cunninghamia lanceolata* (100%) | 20° | West | 0.8 | 200 |
|  | Replicates 2 | *Cunninghamia lanceolata* (100%) | 19° | West | 0.8 | 200 |
|  | Replicates 3 | *Cunninghamia lanceolata* (100%) | 24° | West | 0.8 | 240 |

**Table S2.** Organic phosphorus-mineralizing microorganisms in the soil networks. PCF: pure Chinese fir forest; MCF: mixed Chinese fir forest; SNF: secondary natural forest.

| **Attribution** | **ASV** | **type** | **Genus** | **Degree** |
| --- | --- | --- | --- | --- |
| SNF | ASV_96489 | Bacteria | *unclassified_Micromonosporaceae* | 28 |
|  | ASV_4813 | Fungi | *Chloridium* | 432 |
|  | ASV_2294 | Fungi | *Chloridium* | 412 |
| MCF | ASV_3725 | Fungi | *Penicillium* | 12 |
|  | ASV_8478 | Fungi | *Saitozyma* | 338 |
|  | ASV_5158 | Fungi | *Saitozyma* | 3 |
|  | ASV_11005 | Fungi | *Talaromyces* | 4 |
|  | ASV_10364 | Fungi | *Saitozyma* | 12 |
| PCF | ASV_69635 | Bacteria | *Burkholderia-Caballeronia-Paraburkholderia* | 9 |
|  | ASV_10130 | Fungi | *Talaromyces* | 243 |

**Figure S1.** Concentrations of various P fractions in the three forests. PCF: pure Chinese fir forest; MCF: mixed Chinese fir forest; SNF: secondary natural forest. The values are means ± SEs (*n* = 3). Different letters indicate statistical significance at *p* = 0.05.


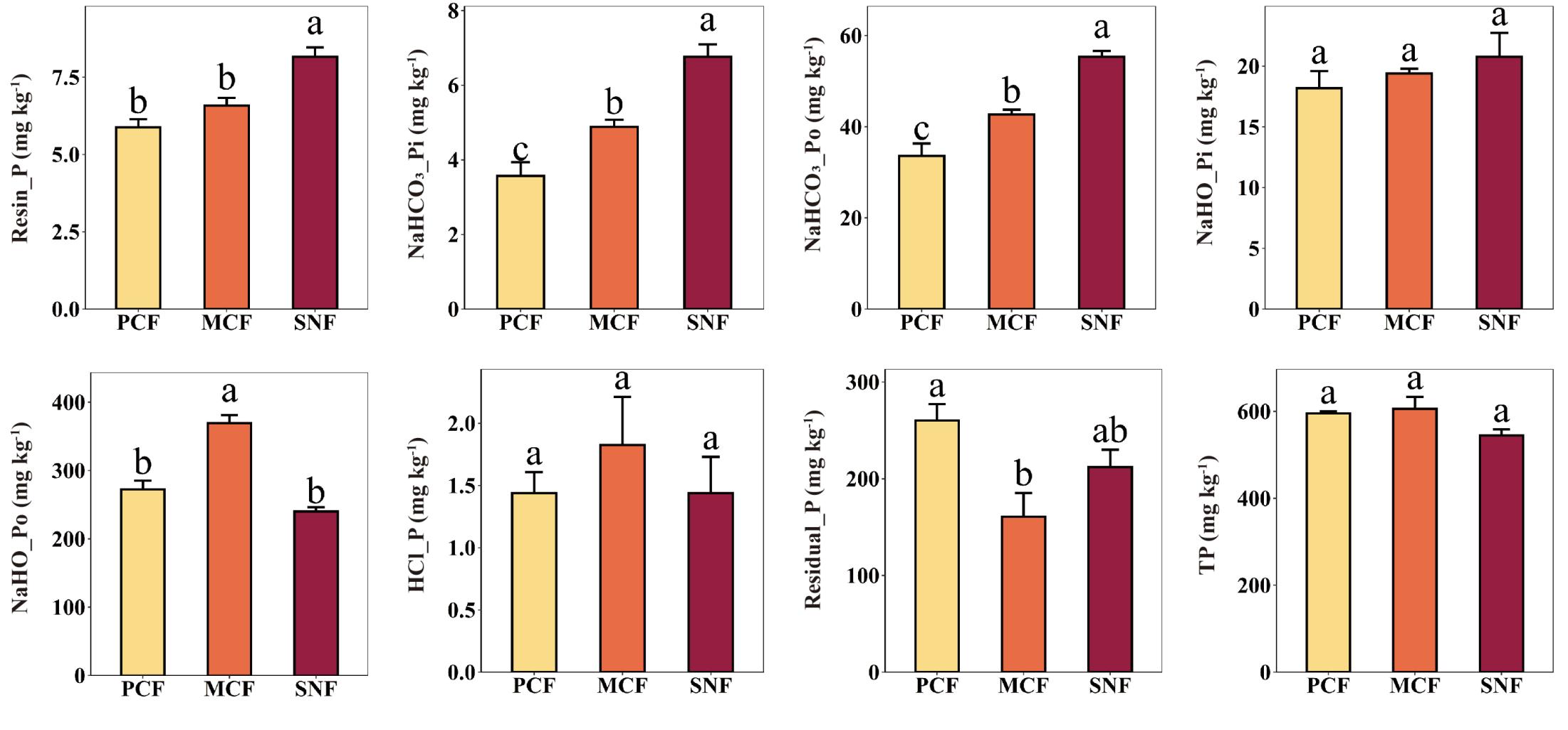


**Figure S2.** The differences in soil microbial community composition among the different forest types. LDA scores showed significant differences in the microbial community among forest types (log LDA > 2.0). PCF: pure Chinese fir forest; MCF: mixed Chinese fir forest; SNF: secondary natural forest.


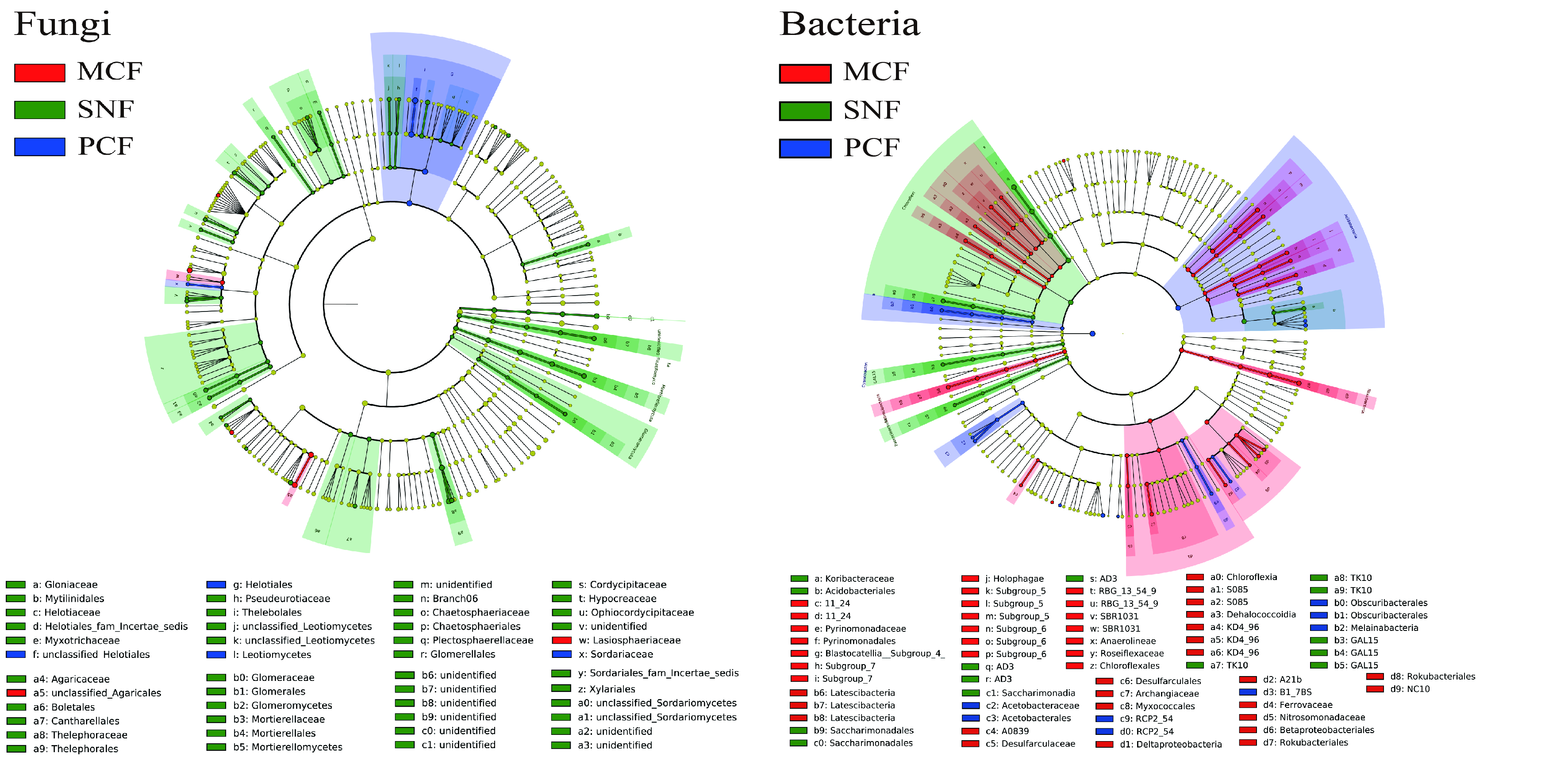


**Figure S3.** Dynamics of microbial alpha diversity during culture. PCF: pure Chinese fir forest; MCF: mixed Chinese fir forest; SNF: secondary natural forest. The values are means ± SEs (*n* = 3). Different letters indicate statistical significance at *p* = 0.05.


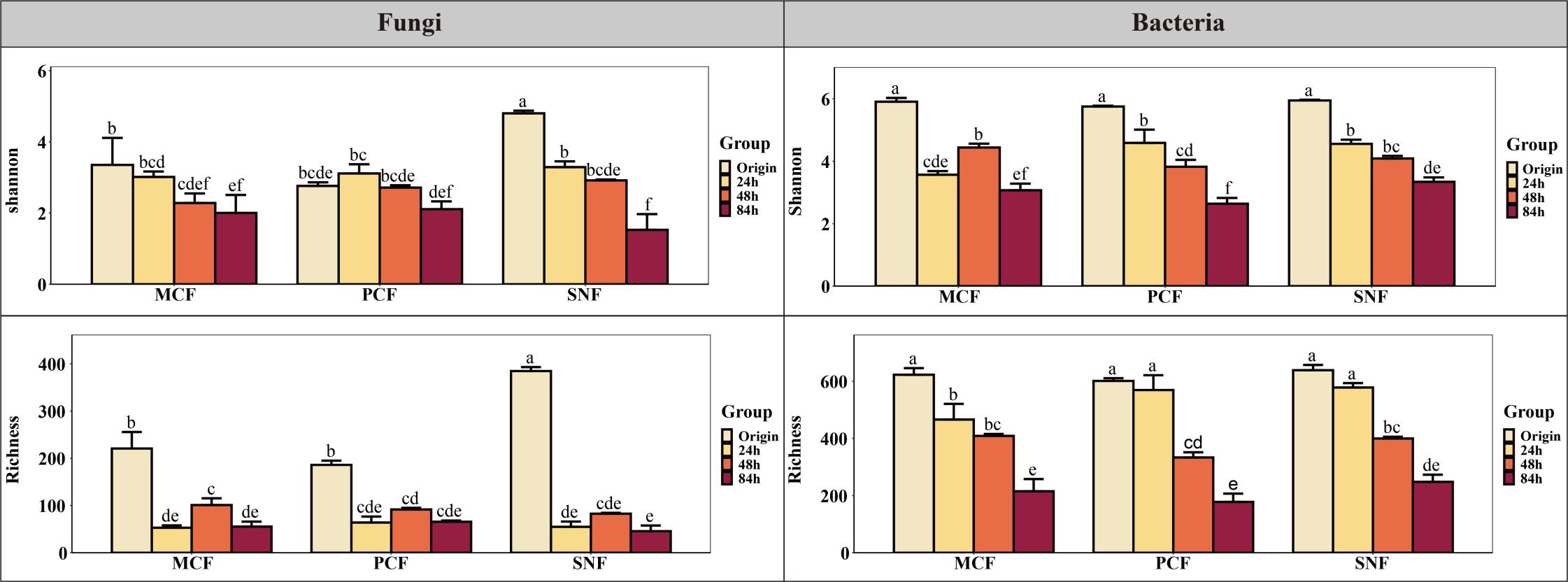


**Figure S4.** Changes of medium pH during culture. PCF: pure Chinese fir forest; MCF: mixed Chinese fir forest; SNF: secondary natural forest; Po: organic phosphorus. Error bars denote the standard error of the mean (*n* = 3).

**
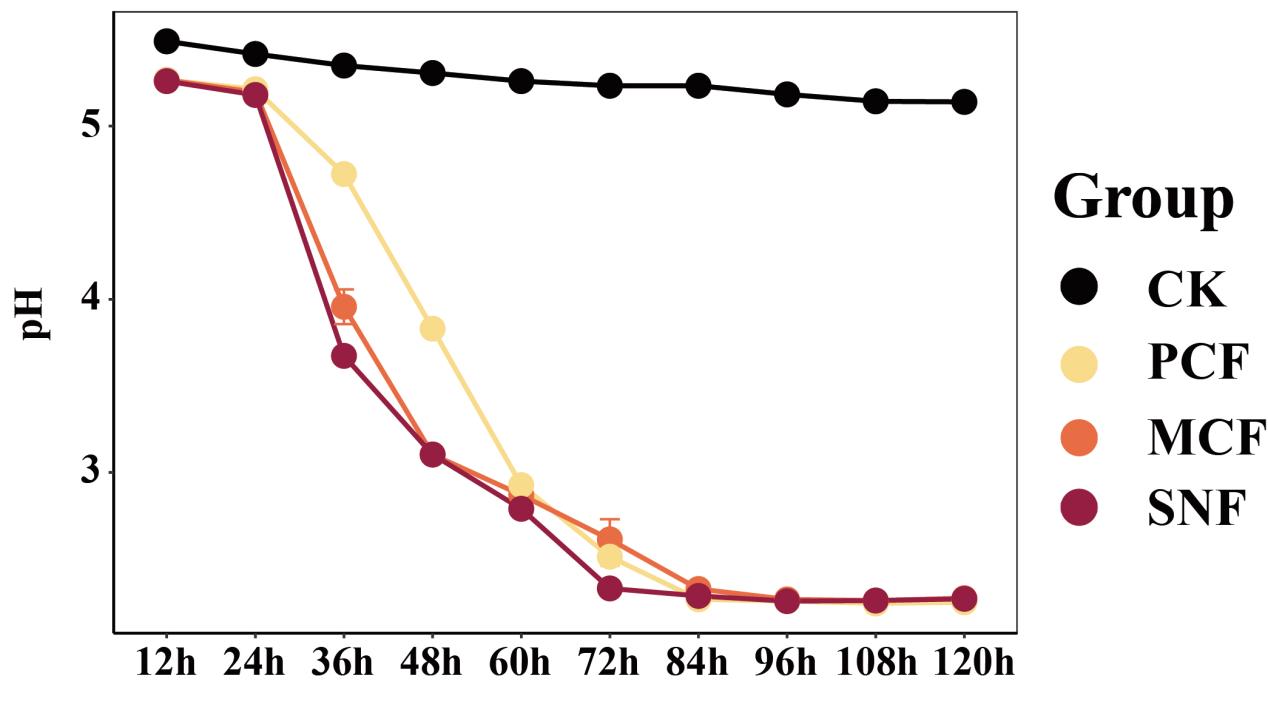
**


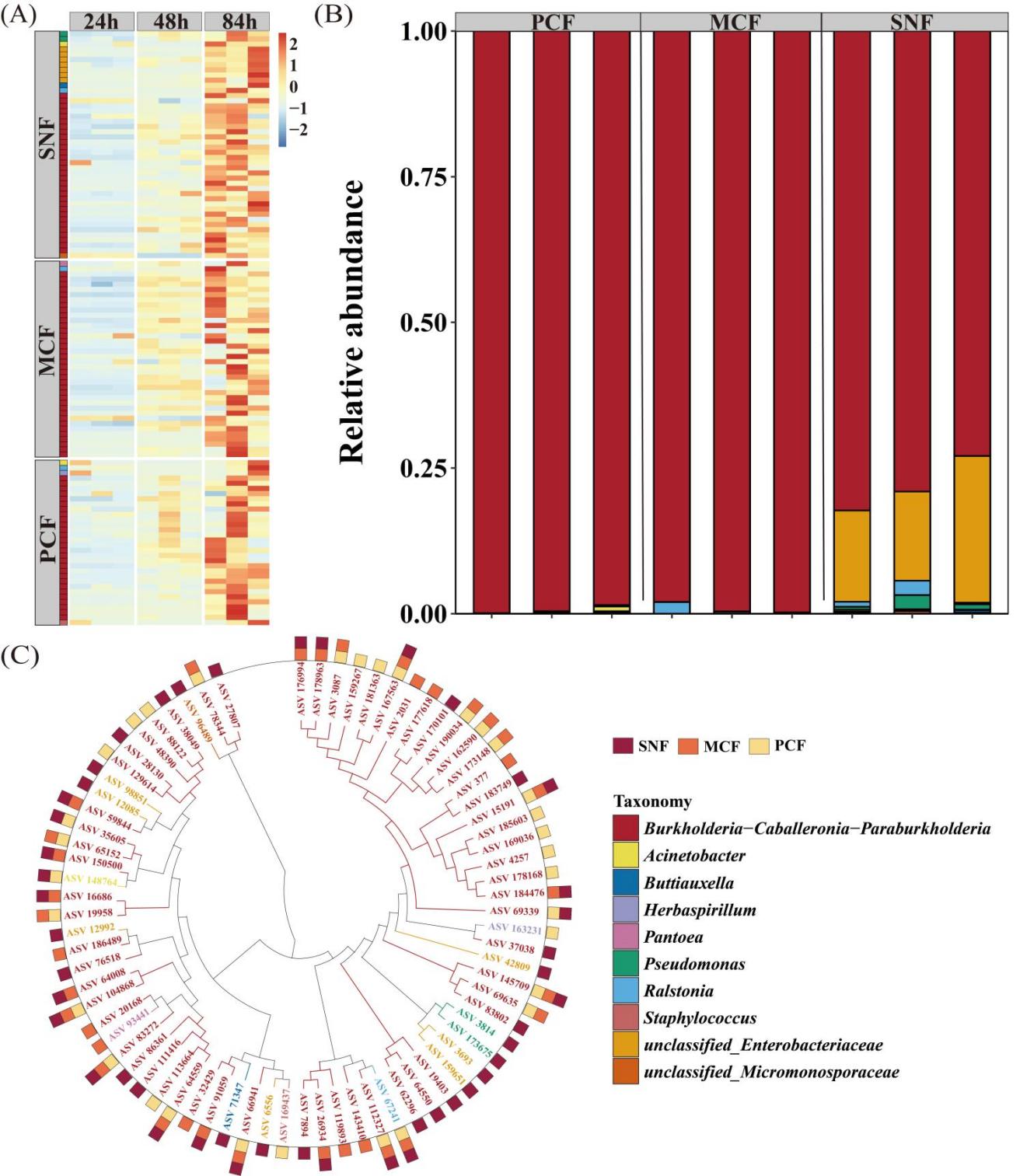
**Figure S5.** Identification of organic phosphorus-mineralizing bacteria. (A) Changes in the relative abundance of organic phosphorus-mineralizing bacteria at the ASV level. (B) Proportions of organic phosphate-mineralizing bacteria at the genus level in different forest types. (C) Phylogenetic relationships among the organic phosphorus-mineralizing bacteria. PCF: pure Chinese fir forest; MCF: mixed Chinese fir forest; SNF: secondary natural forest.

**Figure S6.** Microbial co-occurrence networks of the cultured fungal communities. The node size indicates the degree of the node. Green nodes indicate organic phosphorus-mineralizing fungi, blue nodes indicate network hubs, and red nodes indicate fungi that were identified as both organic phosphorus-mineralizing fungi and network hubs. A blue edge indicates negative interactions, while a red edge indicates positive interactions. PCF: pure Chinese fir forest; MCF: mixed Chinese fir forest; SNF: secondary natural forest.


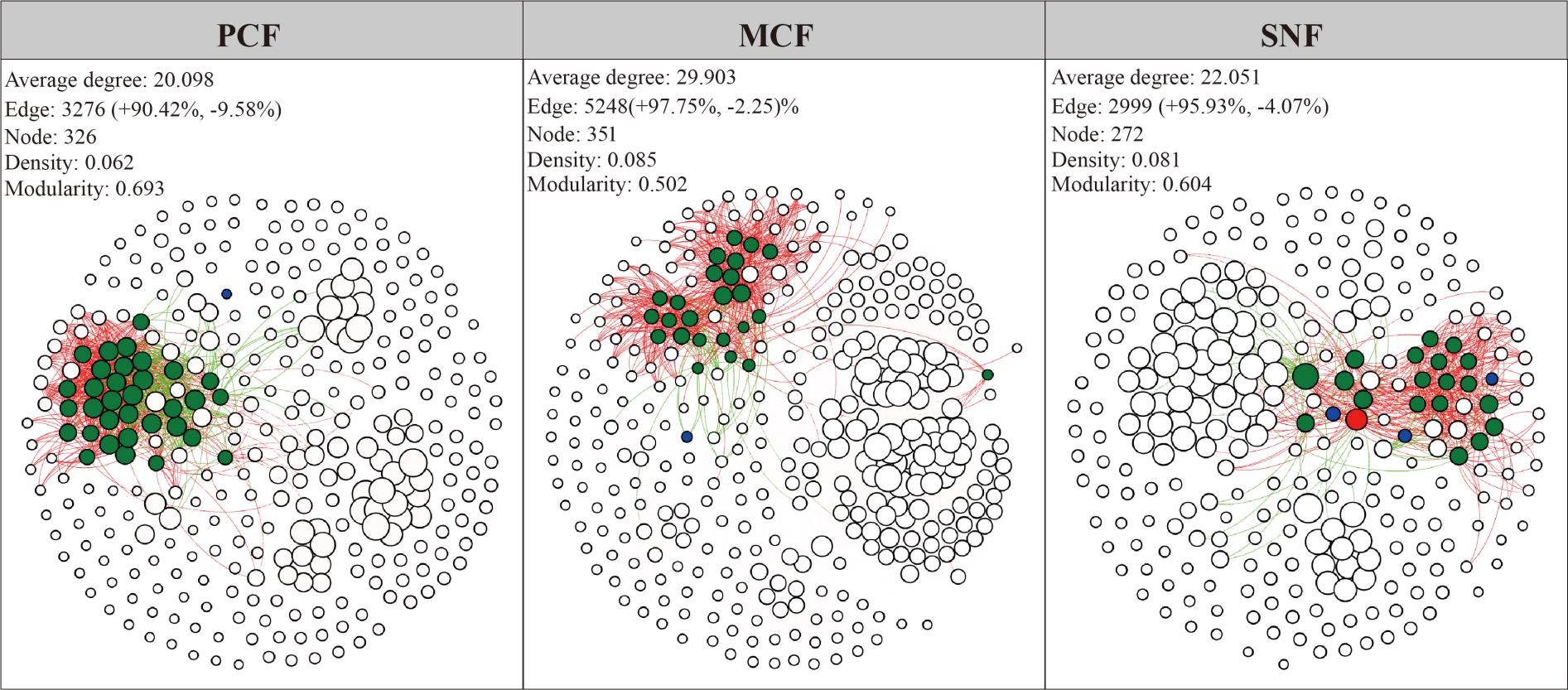


**Figure S7.** Microbial co-occurrence networks of the cultured bacterial community. Node size indicates the degree of the node. Green nodes indicate organic phosphorus-mineralizing bacteria, blue nodes indicate network hubs, and red nodes indicate bacteria that were identified as both organic phosphorus-mineralizing bacteria and network hubs. A blue edge indicates negative interactions, while a red edge indicates positive interactions. PCF: pure Chinese fir forest; MCF: mixed Chinese fir forest; SNF: secondary natural forest.


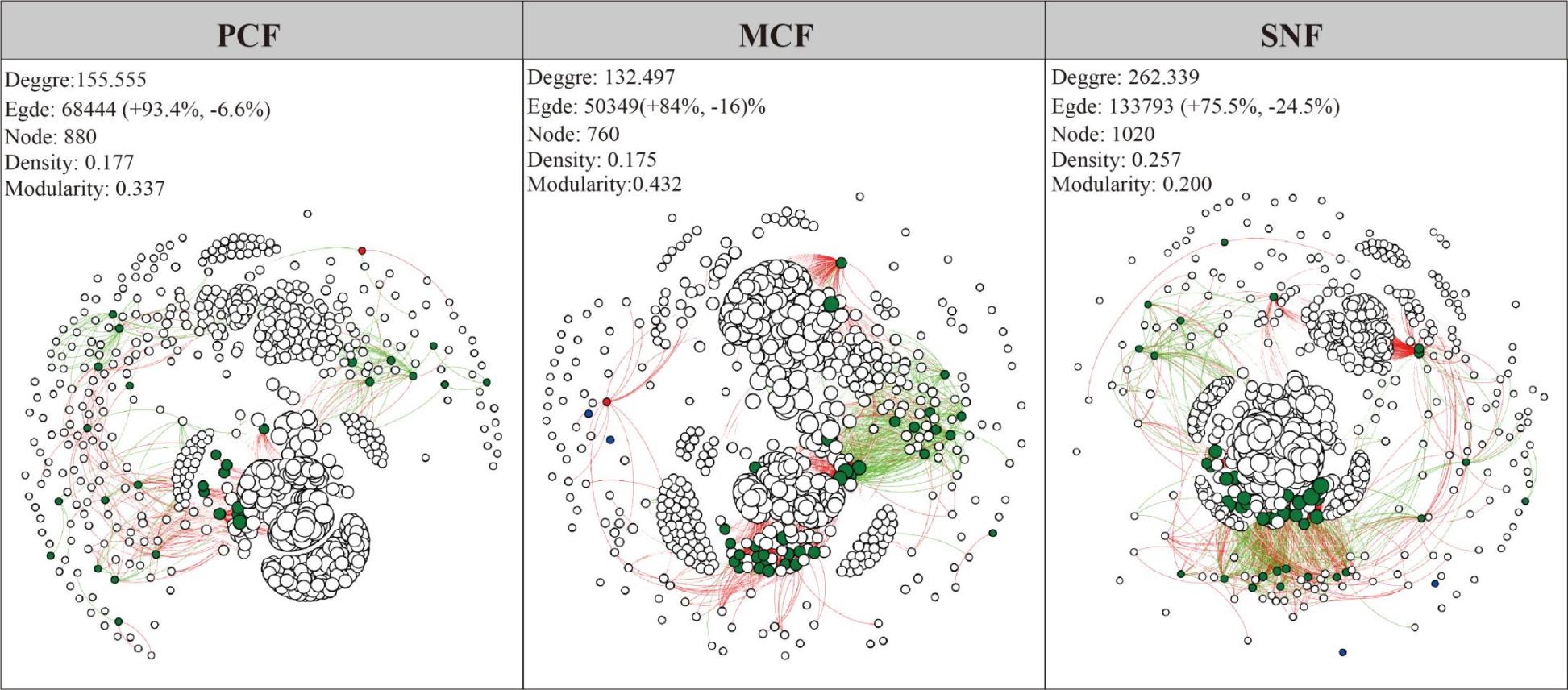


**Figure S8.** Dynamics of acid phosphatase (ACP) activity in cultures of soil from the three forest types at a dilution ratio of 100000. PCF: pure Chinese fir forest; MCF: mixed Chinese fir forest; SNF: secondary natural forest. The error bars denote the standard error of the mean (*n* = 3).


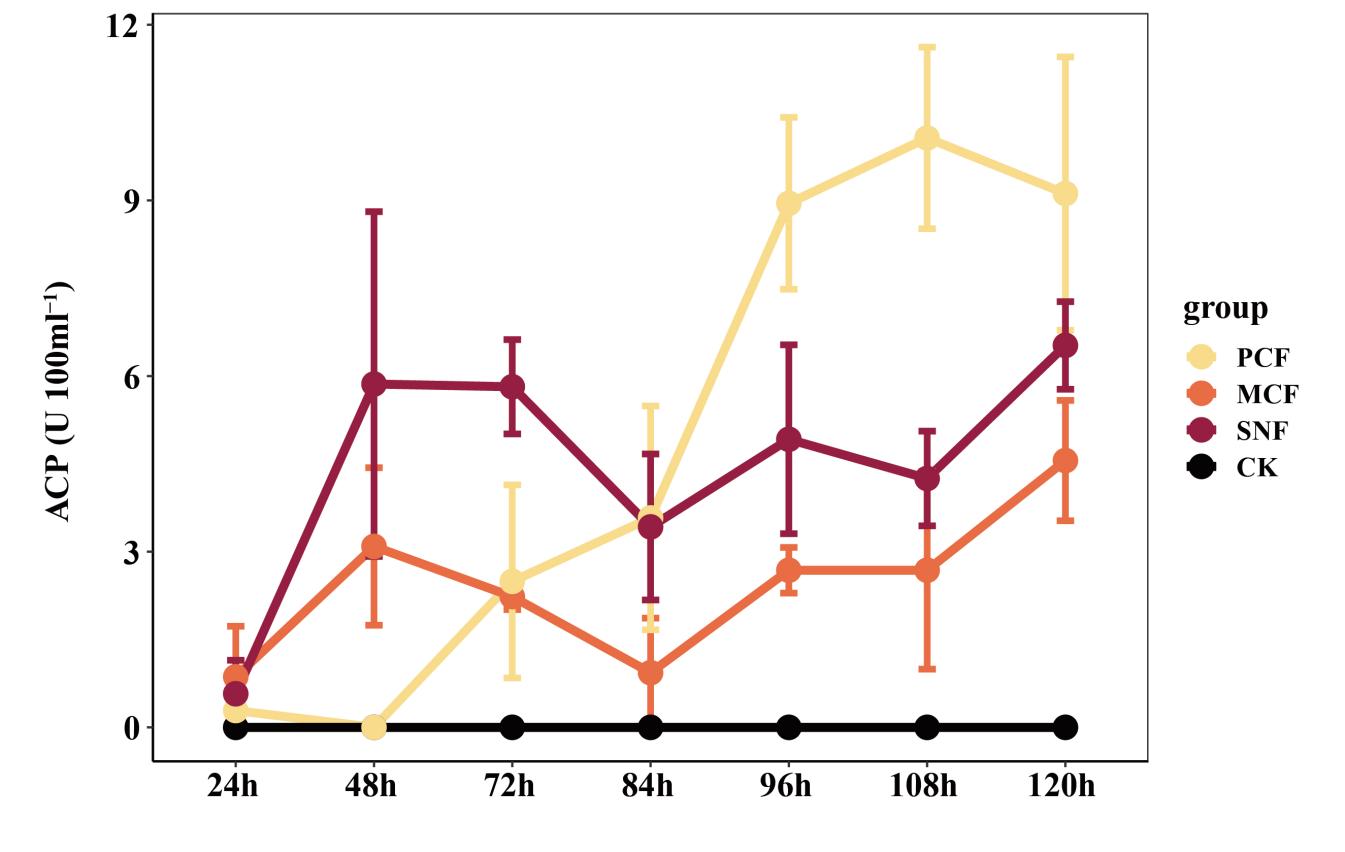

Supplement: Supplementary tables and figures — This file contains 2 supplementary tables and 8 supplementary figures. [file spectrum.01355-23-s0001.docx]
